# Treatment of adult patients with a humeral shaft fracture

**DOI:** 10.2340/17453674.2026.45597

**Published:** 2026-03-17

**Authors:** Thomas IBOUNIG, Olof WOLF, William M OLIVER, Dennis KARIMI, Bjarke VIBERG, Maire RATASVUORI, Antti P LAUNONEN, Tuomas LÄHDEOJA, Jeppe V RASMUSSEN, Lasse RÄMÖ

**Affiliations:** 1 Finnish Centre for Evidence-Based Orthopaedics (FICEBO), Department of Orthopaedics and Traumatology, University of Helsinki and Helsinki University Hospital, Helsinki, Finland; 2 Department of Surgical Sciences, Orthopaedics, Uppsala University and Department of Orthopaedics and Hand Surgery, Uppsala University Hospital, Uppsala, Sweden; 3 Mass General Brigham Orthopedics & Sports Medicine, Wentworth-Douglass Hospital, Dover, NH, USA; 4 Trauma Orthopaedic Research Copenhagen Hvidovre (TORCH), Department of Orthopaedic Surgery, Copenhagen University Hospital Hvidovre, Denmark; 5 Department of Orthopaedic Surgery and Traumatology, Odense University Hospital, Denmark; 6 Department of Orthopaedics, South Karelia Central Hospital, Lappeenranta, Finland; 7 Department of Orthopaedic Surgery, Tampere University Hospital, Tampere, Finland; 8 Department of Orthopaedic Surgery, Herlev and Gentofte Hospital and Department of Clinical Medicine, University of Copenhagen, Denmark

## Abstract

This educational review outlines the core principles of humeral shaft fracture (HSF) management and is designed for orthopedic trainees, general orthopedic surgeons, emergency physicians, and allied health professionals who participate in the acute or postoperative care of patients with HSFs. The content integrates the authors’ expert opinion with the current evidence. Humeral shaft fractures account for 1–3% of adult fractures, most often resulting from low-energy falls in older adults or high-energy trauma in younger patients. Although open fractures and neurovascular injuries are rare, primary radial nerve palsy (RNP) occurs in about 10% of cases. Diagnosis relies primarily on clinical evaluation and standard radiographs, with CT or MRI reserved for complex or pathological cases. Functional bracing has traditionally been the mainstay of nonsurgical treatment, achieving good long-term results but with nonunion rates up to 25%. Surgical fixation methods—including open reduction and internal fixation, minimally invasive plate osteosynthesis, and intramedullary nailing—allow earlier mobilization and more predictable fracture union but carry risks of iatrogenic RNP and infection. Management of primary RNP remains largely nonsurgical, with over 90% recovering spontaneously. Nonunion is frequently symptomatic and managed most often with compression plating. Surgery offers faster early recovery and lower nonunion rates, although long-term outcomes converge with successful bracing. Cost-effectiveness analyses suggest surgery may be more economical when productivity loss is considered, particularly for working-age patients. Optimal treatment selection depends on patient age, activity level, fracture characteristics, and patient preference, emphasizing shared decision-making.

Box 1. Conclusion

- The authors suggest a treatment strategy for HSF in Figure 3, and we emphasize shared decision-making when deciding on the treatment method.
- Both surgical and nonsurgical treatments provide comparable long-term outcomes.
- Surgery leads to faster functional recovery and may be a viable option for patients prioritizing minimal absence from heavy physical work or hobbies but carries a higher risk of iatrogenic RNP or infection.
- Nonunion is the most common complication of nonsurgical treatment and is associated with impaired longer-term patient-reported outcomes, even when union is successfully achieved following nonunion surgery.
- The choice between ORIF and IMN should be individualized based on patient factors, fracture characteristics, and surgeon preference.

## Epidemiology

Humeral shaft fractures (HSFs) comprise 1–3% of adult fractures and 13% of humeral fractures with an annual incidence of 10–30 per 100,000 person-years, rising sharply with age—exceeding 100 per 100,000 among those ≥ 80 years [1-3]. Open fractures are rare (2–6%) [1,3,4], and severe neurovascular injuries are uncommon [5,6].

HSFs show a bimodal age distribution: high-energy trauma in young men and low-energy falls in older women, with overall even sex distribution, but 78% of the HSFs occur in people over 50 years [2,7]. In a Finnish cohort, the median age was 61 years for women and 50 years for men. Low-energy falls caused 62% of fractures, followed by traffic accidents (8%), sports injuries (8%), falls from height (3%), arm wrestling (3%), and equestrian accidents (3%). Midshaft fractures were the most common (46%), followed by proximal (34%) and distal shaft fractures (20%). AO/OTA type A was the most prevalent fracture type (48%), followed by type B (36%) and type C (9%). Most of the fractures (93%) resulted from trauma in patients without prior implants or underlying bone pathology. Periprosthetic and pathological fractures comprised 3% and 4%, respectively [3]. The 1‑year mortality was 9.2% overall, 7.0% in non‑pathological fractures, and 58% in pathological fractures [3]. Overall, primary radial nerve palsy (RNP) is the most common associated injury (10–12%) in HSFs [3,8,9].

## Fracture classification

**Figure 1:**
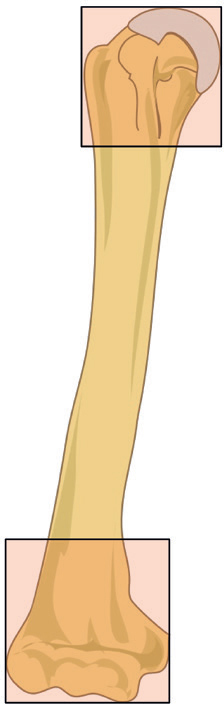
The humeral shaft is defined, using Heim’s square system, as the area between the 2 squares i.e., the area between the proximal and distal epiphyseal regions.

Fracture patterns are influenced by muscle attachments at their respective insertion sites [10] and are most commonly classified using the AO/OTA system [11]. An HSF is defined as a fracture between the proximal and distal end segments of the humerus, which are determined using Heim’s square system—each segment enclosed in a square equal to the width of the epiphysis (Figure 1). Fractures are divided into 3 main types: (A) simple, (B) wedge, and (C) multifragmentary, with each further subdivided into subtypes (1–3) and categorized by location as proximal, middle, or distal 3rd [11].

Box 2. Pearls and pitfalls

- Successful bracing requires proper brace handling, including tightening as needed, and guidance by a skilled physiotherapist during the first weeks.
- Identify patients at risk of developing nonunion as early as 6 weeks, and at the latest at 12 weeks.
- Use an anterior/anterolateral approach for proximal and mid-shaft fractures; a posterior approach for mid-shaft and distal 3rd fractures.
- Gentle handling, adequate exposure, and release of the radial nerve from the intermuscular septum can help reduce the risk of iatrogenic RNP.
- Nonunion following surgical treatment can be related to occult low-grade infection, highlighting the need for intraoperative tissue culture.
- Check for hidden extensions: look carefully for hairline fracture lines (especially with oblique or comminuted patterns) extending proximally or distally.
- Plate length and fixation: use sufficiently long plates, ensuring 6–8 cortices of fixation on each side of the fracture.

The classification demonstrates moderate inter-observer and substantial intra-observer reliability among orthopedic surgeons [12], though its clinical utility is limited, and the description of fracture characteristics, such as location and comminution, is often preferred in daily practice. Proximal 3rd fractures can be difficult to categorize, as about 50% of proximal spiral fractures extend into the humeral head, but the center of the fracture defines location [13].

## Diagnostics

### Clinical presentation

The clinical presentation varies depending on the fracture location. Proximal HSFs typically present with shoulder pain, swelling, and restricted shoulder motion, and patients often support the injured arm against the torso to alleviate discomfort. Mid-shaft fractures are commonly characterized by localized pain, deformity, and hematoma, and may show visible angulation or shortening of the upper arm. Distal fractures may lead to elbow deformity or joint effusion.

Neurological injury is an important consideration after HSFs, with the radial nerve at particular risk in midshaft fractures due to its course in the radial groove [3]. In the distal 3rd, the Holstein–Lewis fracture—a simple spiral pattern with an apex-radial and varus deformity—is notable for its up to 20% risk of RNP [14,15]. RNP typically presents with wrist drop and sensory loss over the dorsal aspect of the hand. In rare cases of proximal HSF, the axillary nerve may be involved, causing deltoid weakness and numbness over the deltoid muscle; this can lead to inferior displacement of the humeral head within the glenoid socket due to loss of deltoid tone, which should not be mistaken for a true shoulder dislocation. Ulnar and median nerve injuries are uncommon and usually occur in distal fractures, potentially resulting in sensory disturbances and impaired hand function, depending on the extent of nerve damage.

### Imaging

Standard orthogonal radiographs, including anteroposterior and lateral views, are the first-line modality and are sufficient in most cases. CT provides detailed bone morphology and 3D reconstruction is useful for complex fractures, suspected intra-articular extension, and preoperative planning. If vascular injury is suspected, CT angiography should be performed. Ultrasound (US) may be helpful to assess the extent of radial nerve injury [16]. MRI is rarely indicated but may help assess soft tissue, nerve involvement, or suspected pathological fractures [17].

### Patient experience

Patients with HSFs typically experience immediate, intense pain and an abrupt loss of upper arm function, often describing the sensation as a complete disconnection from the limb. In the days following the injury, severe pain, marked fracture mobility, swelling, and discoloration are common, making even the most basic activities of daily living (ADLs) extremely challenging across all age groups. This acute phase is frequently remembered as profoundly distressing. As healing progresses, pain subsides, mobility improves, and patients gradually regain independence in ADLs, with a pivotal milestone being the renewed sense of connection to the arm [18]. However, persistent weakness remains common, and patients who do not experience progress in their symptoms tend to change their preference towards surgery.

## Treatment considerations

HSFs have long been considered the type of long bone fracture that can be successfully treated non-surgically with good outcomes [19]. However, surgery offers the advantage of stable fixation, allowing early joint mobilization and restoration of humeral alignment and rotation. Commonly accepted indications for primary surgery include vascular injury, progressive nerve palsy, open fractures, floating elbow, polytrauma, and pathological fractures [20-24]. The authors’ experience is that surgery may also be considered in cases with substantial displacement, threatened skin integrity, high activity demands, intra‑articular extension of the fracture, or segmental fracture patterns.

### Nonsurgical treatment

Functional bracing for HSFs was introduced by Sarmiento et al. [19] in 1977, showing excellent results with a 98% union rate in a series of 51 patients. Since then, the functional brace has become the standard for nonsurgical treatment. However, evidence comparing different nonsurgical methods is limited. Functional braces, hanging casts, and coaptation splints may be similarly effective [25]. Some advocate initial use of a coaptation splint, hanging cast, or collar-and-cuff for 1–2 weeks to allow edema to subside, followed by bracing.

The functional brace can be applied in the emergency department, with adjustments made at the first follow-up if needed. For distal fractures, the brace can be extended to include the condyles of the distal humerus. Patients should be informed that pain can be expected for 3–4 weeks and advised to use pain medication in accordance with local practice. An essential component of successful bracing is patient education, which is often best accomplished by a physiotherapist to ensure that the patient understands and performs exercises given, such as pendulum exercises of the shoulder, free elbow and wrist motion, and use of the hand [26]. The brace should be tightened as the swelling resolves but it can be taken off when taking a shower, and the arm can be placed alongside the body.

### Follow-up scheme

Follow-up at 1–2 weeks includes radiographs to assess displacement, evaluation of pain and skin condition, and assessment of brace tolerance with adjustment if needed. Healing and functional recovery should be assessed at 6- and 12-week follow-ups. The brace is typically maintained until these assessments are complete and may be discontinued once adequate clinical healing is confirmed. Surgical intervention should be considered if persistent pain, fracture-site mobility, or incomplete radiographic healing is observed (see “Delayed nonunion prediction” below). This approach, while not yet supported by high-level evidence, is under investigation in an ongoing RCT [27].

### Surgical treatment

Common surgical techniques for HSFs include open reduction and internal fixation (ORIF), minimally invasive plate osteosynthesis (MIPO), and intramedullary nailing (IMN) [7]. External fixation is rarely indicated [20-24]. Given the limited comparative evidence on fixation methods for HSFs across fracture patterns, the authors note that surgeon familiarity with the chosen technique is an important determinant of method selection.

### Plate osteosynthesis

*ORIF* is the most common surgical technique for HSFs [7,28]. Patient positioning depends on fracture location and surgical approach. Supine or beach chair positions are preferred for proximal and midshaft fractures treated via anterior or anterolateral approaches. Distal fractures requiring radial nerve mobilization are often approached posteriorly, with the patient in either the prone or the lateral decubitus position. Direct lateral or medial approaches are used selectively for specific neurovascular access [29]. After reduction and temporary fixation, a standard 4.5 mm narrow locking compression plate is typically applied, with 3–4 screws (6–8 cortices) on each side of the fracture. Simple fractures benefit from compression plating or lag screws, while comminuted patterns are treated with bridge plating using longer plates [30]. If nerves are exposed (e.g., the radial nerve posteriorly), their position relative to the implant should be documented to prevent complications during potential future removal.

*MIPO* is a less commonly used technique for HSFs [31]. Patients are positioned supine or in the beach chair position using two 3–5 cm incisions placed proximally between the biceps and deltoid/cephalic vein and distally between the biceps and brachialis, avoiding radial nerve exposure [31]. The plate is passed proximal to distal, alignment restored, rotation confirmed, and 3 screws inserted per fragment [32].

### Intramedullary nailing

Intramedullary nailing (IMN) can be performed via an antegrade or retrograde approach [33]. For antegrade nailing, patients are positioned in the beach chair or supine position. Although C‑arm positioning varies across institutions, the authors favor a contralateral approach because it provides consistent axial, AP, and lateral visualization without requiring C‑arm repositioning. Before draping, the surgeon confirms that the patient’s head does not obstruct nail insertion. Retrograde nailing is performed with the patient prone or in lateral decubitus.

The authors’ preferred method for antegrade approach is a 2–3 cm anterolateral incision at the acromial corner. The deltoid is split and the supraspinatus incised in line with its fibers. Entry through the humeral head is performed in extended arm position and confirmed fluoroscopically to minimize iatrogenic cartilage damage. A guidewire is inserted, canal reaming performed if needed, and the nail locked proximally with a jig and distally freehand before repairing the cuff and deltoid.

Retrograde nailing uses an eccentric entry in the dorsal cortical triangle avoiding elbow capsule violation [33]. Through a 4–5 cm incision with longitudinal triceps tendon split, the olecranon fossa is revealed [34]. Careful reaming and insertion help prevent supracondylar fractures. Once the nail is advanced proximally, distal and proximal interlocking are performed as in antegrade nailing, and the triceps fascia is repaired [35].

### External fixation

External fixation has a limited but important role in managing HSF, primarily in cases of open fractures with significant soft tissue or bone loss, polytrauma (as part of damage control), and infected nonunion with associated skin or wound problems [36]. Additional indications include vascular injury and burns. In acute settings, it serves as a limb- or life-saving measure. Humeral pins should be inserted laterally under direct visualization, preferably through a single large incision, to minimize the risk of radial nerve injury. In the forearm, dorsolateral ulnar pins can be placed through stab incisions. In rare cases, external fixation may serve as definitive treatment when further surgery is not feasible [37].

### Follow-up scheme

Given the limited evidence on optimal rehabilitation, the authors suggest following the protocol of an ongoing trial [27]. Physiotherapy may start immediately, with unrestricted but unloaded active motion during the first 2 weeks. Lifting is permitted but limited to light objects (e.g., a bottle of milk), with precautions taken to protect the surgical site during wound healing. From weeks 2 to 6, therapy should maintain unrestricted motion while gradually introducing loading, provided it remains within the pain threshold. After 6 weeks, provided that radiographs show no evidence of healing complications, patients may advance to full loading and unrestricted movement, with physiotherapy aimed at regaining strength and achieving full range of motion.

### Adverse events

The most common adverse events associated with HSF management are non-union, RNP, and infections (Table 1). Based on current literature, 5–9 surgeries are needed to prevent 1 non-union while 13–50 surgeries result in 1 infection or 1 iatrogenic RNP (Table 2) [38-41].

**Table 1:** Most common adverse events in humeral shaft fracture management

Management Adverse event	Proportion (%), [ref.]	Prevention/tips and tricks
**Nonsurgical**		
Nonunion	16–17 in RCTs [38–40]	Proper brace management
	20–23 in clinical cohorts [41]	
Primary radial nerve palsy	12 [8,9]	> 90% recover spontaneously over an average of 19 weeks
Secondary radial nerve palsy	< 1	Early exploration often warranted
Infection	< 1	Close monitoring of the skin during bracing, especially in patients with cognitive impairment
**Surgical**		
Infection	4–9, mostly superficial [39,40]	Deep infections are rare and may be prevented through meticulous soft tissue handling and timely antibiotic prophylaxis
Iatrogenic radial nerve palsy	3–8 [38-40]	Gentle tissue handling, adequate exposure, and release of the nerve from the intermuscular septum; over 90% recover spontaneously. Exploration warranted if suspicion of nerve entrapment under the plate or in the fracture gap
Nonunion	1–5 [38-40]	Rule out occult low-grade infection via intraoperative tissue cultures
Shoulder impairment	13 after IMN [82]	Avoid nailing in patients with healthy rotator cuff
	1–2 after plating	

**Table 2:** Numbers needed to treat (NNT) or harm (NNH) in humeral shaft fracture management

Outcome	Comparison	Estimate	Interpretation
Nonunion prevention			
RCT population	Early surgery vs nonsurgical	NNT = 6–9 [38-41]	6–9 surgeries prevent 1 nonunion
general population	Early surgery vs nonsurgical	NNT = 5–6 [38-41]	5–6 surgeries prevent 1 nonunion
Radial nerve injury	ORIF vs nonsurgical	NNH = 14–50 [38-40]	1 additional nerve injury per 14–50 ORIFs
Infection	ORIF vs nonsurgical	NNH = 13–33 [38,39]	1 additional infection per 13–33 ORIFs
Shoulder impairment	Intramedullary nailing vs ORIF	NNH = 8–9 [82]	1 additional shoulder impairment per 8–9 IM nailings

## Comparison of treatment outcomes

### Nonsurgical treatment vs surgical treatment

**Figure 2:**
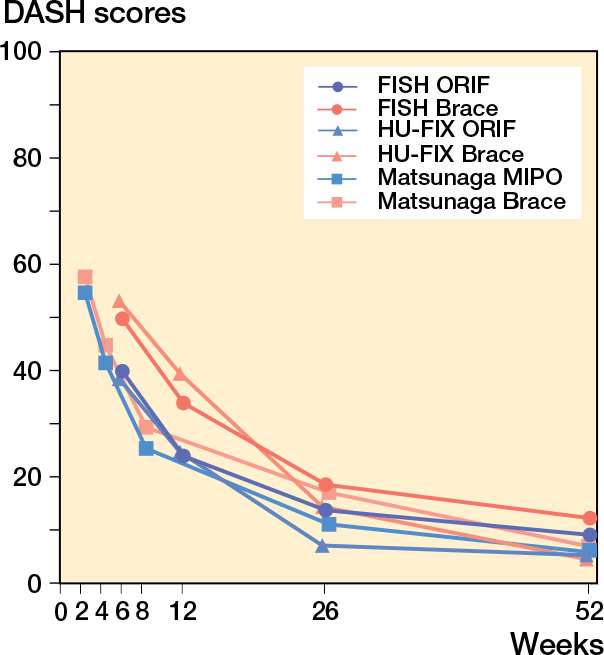
DASH scores in the 3 RCTs comparing surgery with bracing.

3 high-quality RCTs have compared surgical fixation with functional bracing for HSFs (Figure 2) [26,42,43].

A Brazilian RCT [42] found better DASH scores at 6 months with minimally invasive bridge plating (10.9 vs 16.9), though the difference was not clinically significant and had resolved by 12 months. However, the nonunion rate was notably higher in the bracing group (15% vs 0%).

The FISH trial from Finland reported no significant difference in DASH at 12 months (8.9 vs 12.0) [26]. Surgery provided faster early recovery, and 30% of braced patients required secondary surgery. At 2 years, this group had significantly worse DASH scores (17.5) compared with those treated with primary surgery (6.8) or successful bracing (6.0) [44]. By 5 years, outcomes had converged across groups [45]. Nonunion rate was 25% in the bracing group vs 0% in the surgery group [44].

The HU-FIX trial from the UK showed improved DASH and quality-of-life scores at 6 weeks and 3 months with surgery, but no differences at 6 or 12 months. Nonunion was more common in the bracing group (18% vs 6%) [43].

In summary, these 3 RCTs suggest that surgery offers faster functional recovery and a lower risk of nonunion but carries a higher risk for iatrogenic RNP or infection. Long-term outcomes are comparable between surgery and successful bracing, but patients requiring secondary surgery after bracing have inferior results for up to 2 years. These findings underscore the importance of patient selection and shared decision-making in choosing initial treatment.

### IMN vs ORIF

A network meta-analysis found that IMN was associated with worse DASH scores than ORIF (mean difference 8.55), and the difference slightly exceeded the minimal clinically important threshold [39]. A Dutch cohort study reported faster recovery of shoulder function with plating, assessed using the DASH and Constant–Murley scores [46].

A recent meta-analysis of 10 RCTs (512 patients) reported no significant differences in nonunion (8.4% IMN vs 6.4% ORIF), reoperation (11.6% vs 7.6%), or RNP (2.8% vs 4.2%). However, IMN showed advantages with lower infection rates (1.2% vs 5.3%), shorter operative time (61 vs 88 minutes), and faster union (10 vs 11.9 weeks) [47].

Overall, while both techniques offer reliable fracture healing, ORIF tends to provide slightly better functional results, whereas IMN offers surgical advantages such as lower infection risk and quicker union. The choice between methods should be individualized based on patient factors, fracture characteristics, and surgeon preference.

### Cost-effectiveness and return to work

A cost-effectiveness analysis based on the FISH trial found surgery to be more cost-effective than functional bracing when considering total costs, largely due to reduced productivity loss from shorter sick leave. However, bracing remained more cost-effective when only direct treatment costs were considered [48]. Similarly, a recent retrospective study from UK [49] showed that routine fixation of humeral shaft fractures is cost-effective.

In the HU-FIX trial, return-to-work rates and timing were similar: 84% of surgical patients and 81% of braced patients resumed work, with median return times of 7.3 and 10 weeks, respectively [43].

Overall, surgical treatment may be preferable for working-age patients with physically demanding jobs to minimize time off work, while functional bracing remains a viable option for those less impacted by work absence or who wish to avoid surgical risks.

## Special considerations

### Radial nerve palsy (RNP)

Management of RNP in association with HSF has been debated for decades, though high-quality evidence is lacking; most insights stem from observational studies and reviews [9,50-55]. Primary RNP occurs at the time of injury—typically from compression or distraction—while secondary palsy arises during treatment, often due to entrapment beneath a plate or excessive traction related to surgery or bracing [53].

The radial nerve has good recovery potential due to its motor function and proximal innervation sites [56]. Over 90% of palsies, including many secondary cases, recover spontaneously within 1 year without surgery [53]. While diagnosing RNP after humeral fractures is typically straightforward, assessing the severity of damage is more challenging. A favorable outcome depends on restoring nerve conduction before irreversible muscle atrophy occurs. However, predicting which injuries will recover remains difficult. Nerve conduction studies are unhelpful within the first 4 weeks [57]. US shows promise but requires further validation [58].

The timing and necessity of surgical exploration remain the subject of debate. There is broad agreement that early surgical exploration is warranted in open fractures, severe displacement (e.g., in high-energy injuries), vascular injury, or delayed-onset RNP during functional bracing [8,55]. Also, a postoperative RNP should prompt surgical exploration if the nerve was not directly visualized or assessed during the initial operation, as postoperative palsy may result from nerve entrapment beneath the plate or between fracture fragments. In primary RNPs, delayed exploration is typically considered between 2 and 6 months after injury without early recovery signs, such as the return of dorsal‑hand sensation (particularly in the first web space) or early motor activity, including radial wrist deviation from brachioradialis or wrist extension from extensor carpi radialis longus [9,55,57]. The decision is nuanced, as some recoveries may occur even after 1 year [52,55]. While awaiting recovery, joint contracture should be prevented through splinting and passive motion.

If recovery fails, surgical options include neurolysis, nerve grafting (e.g., using the sural nerve for large gaps or neuromas), nerve transfers, and tendon transfers [59,60]. Nerve transfers (e.g., from the motor branch of the flexor digitorum superficialis to the extensor carpi radialis brevis [ECRB] or the motor branch of the flexor carpi radialis [FCR] to the posterior interosseous nerve) are preferred in earlier reconstructions due to their potential for finer motor control, while tendon transfers (e.g., pronator teres to ECRB and FCR to finger extensors) remain more reliable in chronic cases or when nerve repair is not feasible [59,61,62].

### Open injury

The treatment of open injuries follows general principles of open fracture management with early administration of antibiotics, proper debridement of devitalized tissue, irrigation, fracture stabilization, and timely soft tissue coverage.

### Nonunion

#### Management of nonunion

Unlike some upper limb fractures (e.g., lateral clavicle, olecranon, or proximal radius) [63-67], nonunion after nonsurgical treatment of HSF is almost always symptomatic—resulting in pain and arm instability—and typically the patient benefits from surgical fixation. The standard approach involves open reduction, debridement of fibrous tissue and sclerotic bone, and stable fixation using a compression plate. High union rates (94–98%) have been reported with this technique, both with [68] and without autologous bone grafting (ABG) [69]. Avoiding routine ABG removes the risk of donor-site morbidity, which affects up to 38% of patients who undergo bone grafting [69].

Complication rates of nonunion surgery are higher than with primary fixation. Postoperative RNP occurs in up to 18% of cases [70,71] and may persist beyond 1 year in nearly 40% of patients [72]. Infection is reported in approximately 11% of cases [73], emphasizing the importance of obtaining intraoperative tissue cultures on a routine basis.

Based on current evidence, compression plating—either with or without ABG, depending on nonunion type and surgeon preference—is the preferred treatment method.

#### Baseline nonunion prediction

While nonunion surgery is generally successful in achieving fracture healing, it may be associated with poorer long-term patient-reported outcomes [44,74]. These findings highlight the potential benefit of identifying patients at high risk of nonunion early, allowing timely surgical intervention to optimize functional recovery. Several baseline factors have been investigated as potential predictors of humeral shaft fracture nonunion (Supplementary Tables S1–S2). Although findings vary between studies, older age, smoking, and proximal 3rd fracture location have repeatedly been associated with an increased risk of nonunion. However, the overall predictive value of baseline factors alone remains limited, and current risk models offer only modest usefulness in clinical decision‑making.

#### Delayed nonunion prediction

Given the limited accuracy of baseline predictors, delayed assessment has been proposed to identify patients at risk of HSF nonunion. Clinical examination can provide valuable prognostic information; fracture site mobility at 6 weeks demonstrates 82% sensitivity and 99% specificity for predicting nonunion [75]. Radiological measures also contribute: in mid-diaphyseal fractures, each additional millimeter of post-bracing fracture gap increases the odds of failure by 40% [76]. The Radiographic Union Score for HUmeral fractures (RUSHU) assesses early callus formation on anteroposterior and lateral radiographs at 6 weeks (Table 3), with a score < 8 predicting nonunion with ≥ 75% sensitivity and ≥ 65% positive predictive value across multiple studies [77-80]. A retrospective cost-utility analysis suggests that offering surgery to patients with RUSHU < 8 may lower treatment costs and improve EQ-5D health-related quality of life [49].

**Table 3:** Overview of Radiographic Union Score for HUmeral fractures (RUSHU)

Score per cortex	Callus
1	Absent
2	Present, non-bridging
3	Present, bridging

Other modalities, such as US, may further aid early identification, particularly in patients with minimal radiographic callus [81].

## Conclusion including authors’ suggested treatment

**Figure 3:**
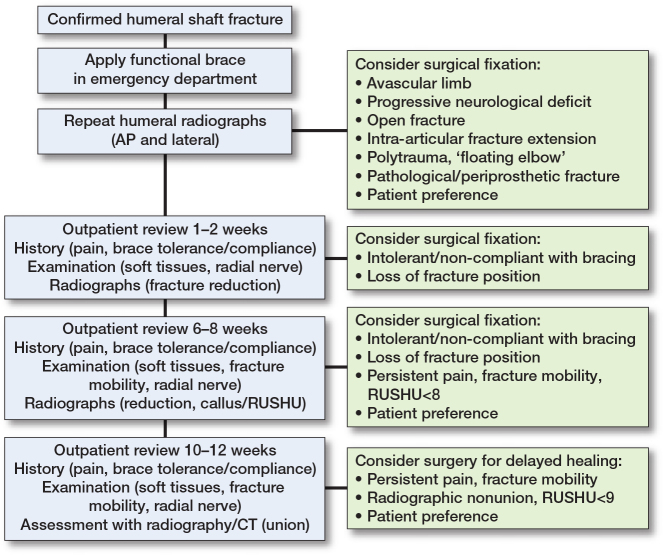
Suggested treatment protocol for humeral shaft fractures.

See Boxes 1–3 and Figure 3.

Box 3. Key topics for future research

- Methods for early identification of patients at risk for nonunion, and the impact of timely surgical intervention on outcomes.
- The role of physiotherapy, particularly in relation to nonsurgical treatment.
- Predicting RNPs that recover spontaneously and those that do not.

### Supplementary data

Tables S1 and S2 are available as Supplememntary data on the article home page, doi: 10.2340/17453674.2026.45597

## Supplementary Material



## Notes

Handling co-editor: Cecilia Rogmark

*Acta* thanks Tore Fjalestad and Hanna Cecilia Björnsson Hallgren for help with peer review of this manuscript.
