# Supplementary material for: Treatment of adult patients with a humeral shaft fracture
Source: Acta Orthop. 2026 Mar 17;97:194–203. doi: 10.2340/17453674.2026.45597 (PMC12994134; doi:10.2340/17453674.2026.45597)
Supplement: Supplementary file 1 [file ActaO-97-45597-s1.pdf]

SUPPLEMENTARY APPENDIX

Treatment of humeral shaft fractures in adults – an Acta educational article

Ibounig et al.

Table of Contents

Table S1. Studies documenting patient-related risk factors for fracture nonunion 2

Table S2. Studies documenting injury-related risk factors for humeral shaft fracture nonunion........ 3

References 4

Table S1. Studies documenting patient-related risk factors for fracture nonunion

Studies in **bold** are specific to humeral shaft fractures.

Factor	Study
Increasing age	**Cox 2000** [1] – after IM nailing<br>**Green 2005** [2]
Female sex	**Neuhaus 2014** [3]<br>**Serrano 2020** [4]<br>**Kim 2023** [5]
Diabetes mellitus	Hernandez 2012 [6]<br>Zura 2016 [7]
Glenohumeral arthritis	**Oliver 2021** [8]
NSAIDs (pre-injury)	Hernandez 2012 [6]<br>**Oliver 2021** [8]<br>Makaram 2021 [9]
NSAIDs (post-injury)	Giannoudis 2000 [10]<br>Burd 2003 [11]<br>**Bhattacharyya 2005** [12]
Bisphosphonates (post-injury)	Solomon 2009 [13]
Smoking	**Green 2005** [2]<br>**Decomas 2010** [14]<br>Hernigou 2013 [15]<br>**Neuhaus 2014** [3]<br>Scolaro 2014 [16]
Alcohol excess	**Foulk 1995** [17]<br>**Olson 2020** [18]<br>**Serrano 2020** [4]
Obesity	**Jensen 1995** [19]<br>**Green 2005** [2]<br>**Decomas 2010** [14]<br>Zura 2016 [7]

Table S2. Studies documenting injury-related risk factors for humeral shaft fracture nonunion.

Factor	Study
Mechanism	Serrano 2020 [4] – pedestrian vs. car
Fracture location	Castella 2004 [20] – proximal<br>Toivanen 2005 [21] – proximal<br>Ekholm 2006 [22] – proximal or middle<br>Rutgers 2006 [23] – proximal<br>Ring 2007 [24] – proximal<br>Broadbent 2010 [25] – proximal<br>Prasarn 2010 [26] – proximal<br>Ali 2015 [27] – proximal
Fracture configuration	Healy 1987 [28] – transverse or oblique<br>Koch 2002 [29] – transverse<br>Castella 2004 [20] – multifragmentary<br>Ring 2007 [24] – oblique or spiral<br>Serrano 2020 [4] – multifragmentary
Fracture displacement	Kim 2023 [5] – anteroposterior angulation, mediolateral translation

References

1. Cox M A, Dolan M, Synnott K, McElwain J P. Closed interlocking nailing of humeral shaft fractures with the Russell-Taylor nail. J Orthop Trauma 2000; 14: 349–53. doi: 10.1097/00005131-200006000-00008

2. Green E, Lubahn J D, Evans J. Risk factors, treatment, and outcomes associated with nonunion of the midshaft humerus fracture. J Surg Orthop Adv 2005; 14: 64–72. PMID: 16115430

3. Neuhaus V, Menendez M, Kurylo J C, Dyer G S, Jawa A, Ring D. Risk factors for fracture mobility six weeks after initiation of brace treatment of mid-diaphyseal humeral fractures. J Bone Joint Surg Am 2014; 96: 403–7. doi: 10.2106/JBJS.M.00089

4. Serrano R, Mir H R, Sagi H C, Horwitz D S, Borade A, Tidwell J E, et al. Modern results of functional bracing of humeral shaft fractures: a multicenter retrospective analysis. J Orthop Trauma 2020; 34: 206–9. doi: 10.1097/BOT.0000000000001666

5. Kim Y J, Taniguchi K, Bowers M R, Lauder A, Parry J A. Initial displacement of humeral shaft fractures Is associated with failure of nonoperative management. J Orthop Trauma 2023; 37: e200–e205. doi: 10.1097/BOT.0000000000002543

6. Hernandez R K, Do T P, Critchlow C W, Dent R E, Jick S S. Patient-related risk factors for fracture-healing complications in the United Kingdom General Practice Research Database. Acta Orthop 2012; 83: 653–60. doi: 10.3109/17453674.2012.747054

7. Zura R, Mehta S, Della Rocca G J, Steen R G. Biological risk factors for nonunion of bone fracture. JBJS Rev 2016; 4. doi: 10.2106/JBJS.RVW.O.00008

8. Oliver W M, Searle H K C, Ng Z H, Molyneux S G, White T O, Clement N D, et al. Factors associated with humeral shaft nonunion. J Shoulder Elbow Surg 2021; 30: 2283–95. doi: 10.1016/j.jse.2021.01.029

9. Makaram N S, Leow J M, Clement N D, Oliver W M, Ng Z H, Simpson C, et al. Risk factors associated with delayed and aseptic nonunion following tibial diaphyseal fractures managed with intramedullary nailing. Bone Jt Open 2021; 2: 227–35. doi: 10.1302/2633-1462.24.BJO-2021-0012.R1

10. Giannoudis P V, MacDonald D A, Matthews S J, Smith R M, Furlong A J, De Boer P. Nonunion of the femoral diaphysis. The influence of reaming and non-steroidal anti-inflammatory drugs. J Bone Joint Surg Br 2000; 82: 655–8. doi: 10.1302/0301-620x.82b5.9899

11. Burd T A, Hughes M S, Anglen J O. Heterotopic ossification prophylaxis with indomethacin increases the risk of long-bone nonunion. J Bone Joint Surg Br 2003; 85: 700–5. PMID: 12892193

12. Bhattacharyya T, Levin R, Vrahas M S, Solomon D H. Nonsteroidal antiinflammatory drugs and nonunion of humeral shaft fractures. Arthritis Rheum 2005; 53: 364–7. doi: 10.1002/art.21170

13. Solomon D H, Hochberg M C, Mogun H, Schneeweiss S. The relation between bisphosphonate use and non-union of fractures of the humerus in older adults. Osteoporos Int 2009; 20: 895–901. doi: 10.1007/s00198-008-0759-z

14. Decomas A, Kaye J. Risk factors associated with failure of treatment of humeral diaphyseal fractures after functional bracing. J La State Med Soc 2010; 162: 33–5. doi:

15. Hernigou J, Schuind F. Smoking as a predictor of negative outcome in diaphyseal fracture healing. Int Orthop 2013; 37: 883–7. doi: 10.1007/s00264-013-1809-5

16. Scolaro J A, Schenker M L, Yannascoli S, Baldwin K, Mehta S, Ahn J. Cigarette smoking increases complications following fracture: a systematic review. J Bone Joint Surg Am 2014; 96: 674–81. doi: 10.2106/JBJS.M.00081

17. Foulk D A, Szabo R M. Diaphyseal humerus fractures: natural history and occurrence of nonunion. Orthopedics 1995; 18: 333–3. doi: 10.3928/0147-7447-19950401-04

18. Olson J J, Entezari V, Vallier H A. Risk factors for nonunion after traumatic humeral shaft fractures in adults. JSES Int 2020; 4: 734–8. doi: 10.1016/j.jseint.2020.06.009

19. Jensen A T, Rasmussen S. Being overweight and multiple fractures are indications for operative treatment of humeral shaft fractures. Injury 1995; 26: 263–4. doi: 10.1016/0020-1383(95)90012-m

20. Castella FB, Garcia FB, Berry EM, Perello EB, Sanchez-Alepuz E, Gabarda R. Nonunion of the humeral shaft: long lateral butterfly fracture--a nonunion predictive pattern? Clin Orthop Relat Res 2004: 227–230. doi:

21. Toivanen J A, Nieminen J, Laine H J, Honkonen S E, Jarvinen M J. Functional treatment of closed humeral shaft fractures. Int Orthop 2005; 29: 10–13. doi: 10.1007/s00264-004-0612-8

22. Ekholm R, Tidermark J, Tornkvist H, Adami J, Ponzer S. Outcome after closed functional treatment of humeral shaft fractures. J Orthop Trauma 2006; 20: 591–6. doi: 10.1097/01.bot.0000246466.01287.04

23. Rutgers M, Ring D. Treatment of diaphyseal fractures of the humerus using a functional brace. J Orthop Trauma 2006; 20: 597–601. doi: 10.1097/01.bot.0000249423.48074.82

24. Ring D, Chin K, Taghinia A H, Jupiter J B. Nonunion after functional brace treatment of diaphyseal humerus fractures. J Trauma 2007; 62: 1157–8. doi: 10.1097/01.ta.0000222719.52619.2c

25. Broadbent M R, Will E, McQueen M M. Prediction of outcome after humeral diaphyseal fracture. Injury 2010; 41: 572–7. doi: 10.1016/j.injury.2009.09.023

26. Prasarn M L, Achor T, Paul O, Lorich D G, Helfet D L. Management of nonunions of the proximal humeral diaphysis. Injury 2010; 41: 1244–8. doi: 10.1016/j.injury.2010.04.002

27. Ali E, Griffiths D, Obi N, Tytherleigh-Strong G, Van Rensburg L. Nonoperative treatment of humeral shaft fractures revisited. J Shoulder Elbow Surg 2015; 24: 210–4. doi: 10.1016/j.jse.2014.05.009

28. Healy WL, White GM, Mick CA, Brooker AF, Jr., Weiland AJ. Nonunion of the humeral shaft. Clin Orthop Relat Res 1987: 206–213. doi:

29. Koch P P, Gross D F, Gerber C. The results of functional (Sarmiento) bracing of humeral shaft fractures. J Shoulder Elbow Surg 2002; 11: 143–50. doi: 10.1067/mse.2002.121634
